# Bacterial over-production of the functionally active human SLC38A2 (SNAT2) exploiting the mistic tag: a cheap and fast tool for testing ligands

**DOI:** 10.1007/s11033-023-08976-3

**Published:** 2024-02-23

**Authors:** Michele Galluccio, Martina Tripicchio, Lara Console, Cesare Indiveri

**Affiliations:** 1 Department DiBEST (Biologia, Ecologia, Scienze Della Terra) Laboratory of Biochemistry, Molecular Biotechnology and Molecular Biology, University of Calabria, Via P. Bucci 4C-6C, 87036 Arcavacata di Rende, Italy; 2 Institute of Biomembranes, Bioenergetics and Molecular Biotechnology (IBIOM), National Research Council (CNR), Via Amendola 122/O, 70126 Bari, Italy

**Keywords:** SNAT2, Proteoliposomes, Refolding, Purification, SLC

## Abstract

**Background:**

SLC38A2 is a ubiquitously expressed Na^+^-dependent transporter specific for small and medium neutral amino acids. It is involved in human pathologies, such as type II diabetes and cancer. Despite its relevance in human physio-pathology, structure/function relationship studies and identification of ligands with regulatory roles are still in infancy.

**Methods and Results:**

The cDNA coding for SLC38A2 was cloned in the pET-28-Mistic vector, and the BL21 codon plus RIL strain was transformed with the recombinant construct. 0.5% glucose and oxygen availability were crucial for protein expression. The over-expressed hSNAT2-Mistic chimera was cleaved on column and purified by nickel-chelating affinity chromatography, with a yield of about 60 mg/Liter cell culture. The purified hSNAT2 was reconstituted in proteoliposomes in an active form with a right-side-out orientation with respect to the native membrane.

**Conclusions:**

The addition of a Mistic tag at the N-terminus of the SNAT2 protein was crucial for its over-expression and purification. The purified protein was functionally active, representing a powerful tool for performing structure/function studies and testing ligands as inhibitors and/or activators.

## Introduction

The SLC38 family includes 11 members, some of which are still not definitively characterized or orphans. According to the old classification, these transporters were named SNATs (Sodium dependent Neutral Amino acid Transporters) and divided into two sub-classes referred to as system A and system N type transporters: the first has broader substrate specificity for small and medium neutral amino acids, the second is specific for larger amino acids and exhibits dependence on H^+^ besides Na^+^ [1, 2]. From a physiological point of view, some of the SLC38 members, such as SLC38A1 and SLC38A2, have also been called "loaders," that is, they are involved in the net uptake of amino acids; others, like SLC38A5, are called "controllers" since they counteract the accumulative power of the loaders [3]; in the case of SLC38A9 a transceptor function has been established, which is crucial for sensing amino acid sufficiency at the lysosomal membrane for modulating mTORC1 [4, 5]. Member 2, SLC38A2, also known as SNAT2 or ATA2, has been considered both a "loader" and "transceptor" [6–8]; in this respect, SNAT2 may regulate its expression depending on amino acid concentration [9]. Indeed, during amino acid starvation, eIF2α phosphorylates the transcription factor ATF4, which in turn increases the transcription of the SNAT2 gene [10]. The hydropathy profile of the SNAT2 protein is characterized by 11 transmembrane domains, with the N-terminal and C-terminal oriented towards the cytoplasm and outside the cell membrane, respectively [11]. It is ubiquitously expressed and involved in cell volume regulation upon hypertonic exposure [12]. Its involvement in human pathologies, such as type II diabetes and cancer, is also documented to some extent [13–18]. Indeed, it is up-regulated in ASCT2(-/-) hepatoma cells, where it works as a glutamine supplier in the place of ASCT2 [19]. A similar behavior is reported in breast cancer, where the hypoxia triggers up-regulation of the SNAT2 transporter to sustain glutamine uptake, which can fuel the TCA cycle via glutaminolysis [14]. This up-regulation causes oxidative stress tolerance and is associated with a worse prognosis in triple-negative breast cancer [17]. For all these reasons, SNAT2 may be considered a pharmacological target [14, 17, 18]. Despite the importance of this transporter in human pathophysiology, it is still poorly characterized in terms of structure/function relationships, and to our knowledge, only one specific inhibitor has been recently proposed [20]. No data on 3D structure of the human or animal orthologs of SNAT2 are available. Some information on the sodium binding site of the rat ortholog have been obtained by bioinformatics, mutagenesis, and voltage clamp analysis [21]. One of the approaches for studying structure/function relationships and testing inhibitors to design potential drugs is the over-expression of transport proteins in *E. coli* and the reconstitution of the functional transporters in proteoliposomes. This approach has never been used for studying hSNAT2 [22]. Moreover, the recombinant hSNAT2 has never been isolated from eukaryotic cells so far. Using bacteria in the place of eukaryotic expression systems gives the advantage of lowering costs and obtaining larger amounts of proteins. However, problems arising in expressing human proteins should be overcome by specific strategies. In this work, the Mistic tag and other tricks have been exploited to successfully over-express the human SNAT2. The protein purified by nickel-chelating affinity chromatography has been reconstituted in proteoliposomes to assess its functionality.

## Material and methods

His Select Nickel affinity gel (P6611), Monoclonal Anti-polyHistidine-Peroxidase antibody (A7058), Amberlite XAD-4 (06444), egg yolk phospholipids, cholesterol (C3045), Thrombin (605,157), n-Dodecyl β-D-maltoside (D4641), Sephadex G-75 (G75120), L-glutamine (G1626) from Merck Life Science; Isopropyl β-D-1-tiogalattopiranoside (IPTG, A4773) from AppliChem; restriction endonucleases, T4 DNA ligase (EL0014), and Phusion™ High-Fidelity DNA Polymerase (F530S) from ThermoFisher scientific; MegaMan Human Transcriptome Library, BL21 codon plus RIL strain from Agilent technologies; Lemo21(DE3) strain from New England Biolabs; C41(DE3) from Lucigen. Pico-Fluor Plus and L-[^3^H]-glutamine from Perkin Elmer; pET-28a-Mistic (#85,999) from Addgene.

## Cloning of human SNAT2

The cDNA encoding hSNAT2 transporter (UniProtKB: Q96QD8) was amplified from MegaMan Human Transcriptome Library using the primers: 5'- GGGAATTCCATATGAAGAAGGCCGAAATGGGACGAT-3' (forward) and 5'- ATAAGAATGCGGCCGCATGGCCACCTCCAGGTGCATTG-3' (reverse), containing the underlined *Nde*I and *Not*I restriction sites, respectively. The cDNA was cloned between *Nde*I and *Not*I restriction sites of the pET-28a-Mistic, obtaining the pET-28a-Mistic-SNAT2-6His constructs, which encodes an N-terminal Mistic-tagged and C-terminal 6His-tagged human SNAT2 protein.

## Expression of human SNAT2 transporter

To produce the hSNAT2, *E. coli* BL21(DE3) codon plus RIL, C41(DE3), and Lemo21(DE3) cells were transformed with the pET-28a-Mistic-SNAT2-6His and selected on LB-agar plates added with 30 µg/mL kanamycin and 34 µg/mL chloramphenicol. LB, TY 2X, or Terrific media in the absence or the presence of 0.5% glucose were tested. One colony was inoculated in 5–10 mL of a specific medium for small-scale experiments and cultured overnight at 37 °C under rotary shaking (160 rpm). The inoculum was divided the day after into two populations, each diluted 1:20 in a fresh selective medium and treated for glucose and IPTG testing. Flasks were filled with indicated medium volumes to test the effect of oxygenation. When the optical density of the culture reached 0.8–1 (at 600 nm), the temperature was lowered to 28 °C for up to 6 h, and different IPTG concentrations (0.05–1 mM) were tested. Every two hours, aliquots were collected and centrifuged at 6,000 × g, 4 °C for 10 min. A bacterial pellet aliquot deriving from 200 mL cell culture was dissolved in 15 mL 20 mM Hepes/NaOH, 300 mM NaCl pH 7.0 and sonified in an ice bath for 5 min (1 s on and 1 s off) using a Branson SFX 550 sonifier at 150 Watt. The cell lysate was centrifuged for 10 min at 13.000 × g, and the pellet obtained was stored at -20 °C or further analyzed.

## Purification, thrombin treatment, and desalting of the hSNAT2 protein

To purify SNAT2, the pellet deriving from 6 mL out of 15 mL of bacterial suspension was washed twice with 0.1 M Tris/HCl pH 8.0 and centrifuged at 15,000 × g for 10 min. The pellet was solubilized by adding 1.6 mL of 8 M urea, 80 µL of 500 mM DTE, 216 µL of 10% sarkosyl, and shaked for 30 min in a fixed angle rotator for tubes. Then, 1 mL of 0.1% sarkosyl, 200 mM NaCl, 10% glycerol, and 20 mM Tris/HCl pH 8.0 (buffer R—refolding) was added and shaked for 30 min. Then, the sample was centrifuged at 15,000 × g for 15 min and at 4 °C, and the supernatant added with 80 µL thrombin (0.1 U/µL), was mixed with 3 mL of His select Ni^2+^affinity gel equilibrated with 5 mL buffer R and shacked for 2 h at 4° C. Then, the resin was packed into a column by a peristaltic pump at 0.3 mL/min. After collecting the passthrough fractions, the column was washed with 6 mL of 0.3% DDM, 200 mM NaCl, 10% glycerol, 5 mM DTE, 20 mM Tris/HCl at pH 8.0 and 15 mL of the same buffer added with 15 mM imidazole. hSNAT2 was eluted with 5 mL of the same buffer with 500 mM imidazole. Fractions of 0.5 mL were collected and analyzed by SDS-PAGE, and the five most abundant were pulled together and loaded onto a PD10 desalting column equilibrated with 0.3% DDM, 10% glycerol, 5 mM DTE, 20 mM Hepes/NaOH at pH 8.0. After loading 1 mL of the same buffer, the protein was eluted with 2.5 mL. In the case of Fig. 4, stronger reducing conditions were maintained, adding 1 M β-mercaptoethanol to the loading buffer. For sarkosyl-PAGE, sarkosyl was used instead of SDS either in loading buffer or in the polyacrylamide solution.

## Western blotting

Recombinant hSNAT2 was immuno-detected using the Monoclonal Anti-polyHistidine-Peroxidase antibody 1:10,000 after 1 h of incubation at room temperature. The reaction was detected by Enhanced Chemi Luminescence (ECL).

## Reconstitution of hSNAT2 in proteoliposomes

Proteoliposomes reconstitution was achieved by mixing 5 µg of desalted hSNAT2, 70 µL of 10% C_12_E_8_, 100 µL of 10% egg yolk phospholipids in the sonicated liposomes form added with 7.5% cholesterol as previously described [23], 10 mM DTE and 20 mM Hepes/NaOH pH 7.5 in a final volume of 700 µL and incubated for 60 min at 23 °C under rotatory stirring (1200 rpm) with 0.5 g of Amberlite XAD-4 resin.

## Transport measurements

After reconstitution, 550 µL of proteoliposomes were passed through a Sephadex G-75 column (0.7 cm diameter × 15 cm height) equilibrated with a 20 mM Hepes/NaOH pH 7.5. Aliquots of 100 µL of the eluate were used for transport assay at room temperature. The uptake was started by 50 µM [^3^H]-glutamine (20 Ci/mmol) in the presence of Na-gluconate at the indicated concentration and stopped at the desired times.

Liposomes not harboring hSNAT2 were used as a control. At the end of the transport reaction, each sample was passed through a Sephadex G-75 column (0.6 diameter × 8 cm height), buffered with 50 mM NaCl to remove the extra-liposomal radioactivity.

A 3 mL aliquot of liquid scintillation cocktail was added to the eluted proteoliposomes for radioactivity counting. Data were corrected by subtracting the control values. Grafit 5.0.13 was used for non-linear regression analysis.

## Results and discussion

### Over-expression of the hSNAT2 transporter

The constructs obtained by cloning SNAT2 cDNA into pET-21a( +), pET-28a( +), and pH6Ex3 were first employed for transforming Lemo21(DE3), C41(DE3), or BL21(DE3) codon plus RIL *E. coli* strains; very small if any protein expression was obtained in all combinations tested, probably due to the scarce solubility of the protein of interest and its toxicity for bacteria. Then, we exploited a strategy for improving the solubility. The pET-28-Mistic plasmid was adopted for introducing the Mistic tag, a small *Bacillus subtilis* protein that, besides improving the solubility of the hydrophobic transporter, also targets the chimeric protein to the membrane [22]. To overcome possible codon usage problems, the Mistic-SNAT2-6His construct was used to transform *E. coli* BL21(DE3) codon plus RIL strain, which supplies tRNA specific for human codons. A colony was inoculated at 37 °C in LB broth and cultured either in the absence or in the presence of 0.5% glucose and at different IPTG concentrations.

**Fig. 1:**
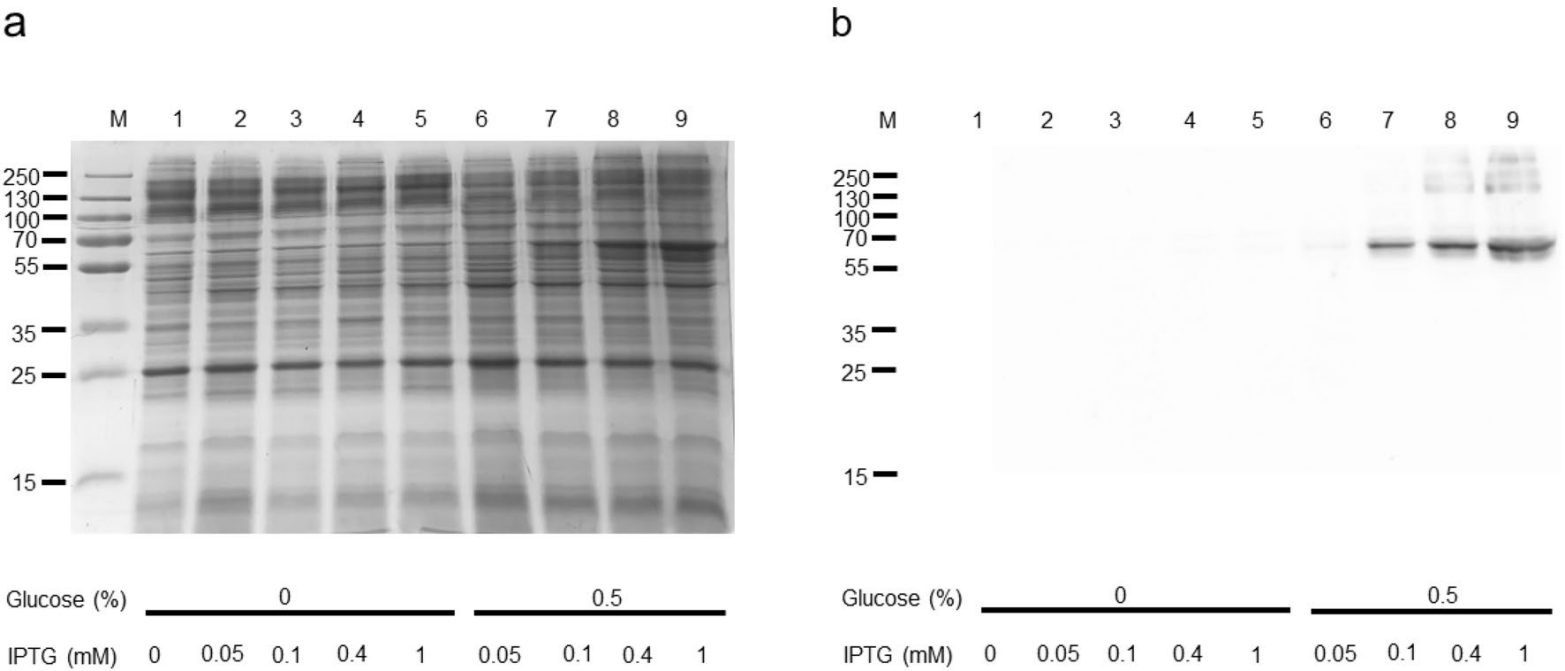
Human SNAT2 expression: the effect of glucose and IPTG *E. coli* BL21(DE3) codon plus RIL was transformed with Mistic-SNAT2 construct and cultured in the absence or presence of 0.5% glucose. The effect of different IPTG concentrations was tested. (a) SDS-PAGE of insoluble fractions: 1, uninduced cell lysate; 2–9 induced cell lysates in different conditions: glucose and IPTG concentration are indicated in the figure; M, page ruler prestained plus marker; (b) Western blotting using anti His antibody of the samples loaded as in (a)

**Fig. 2:**
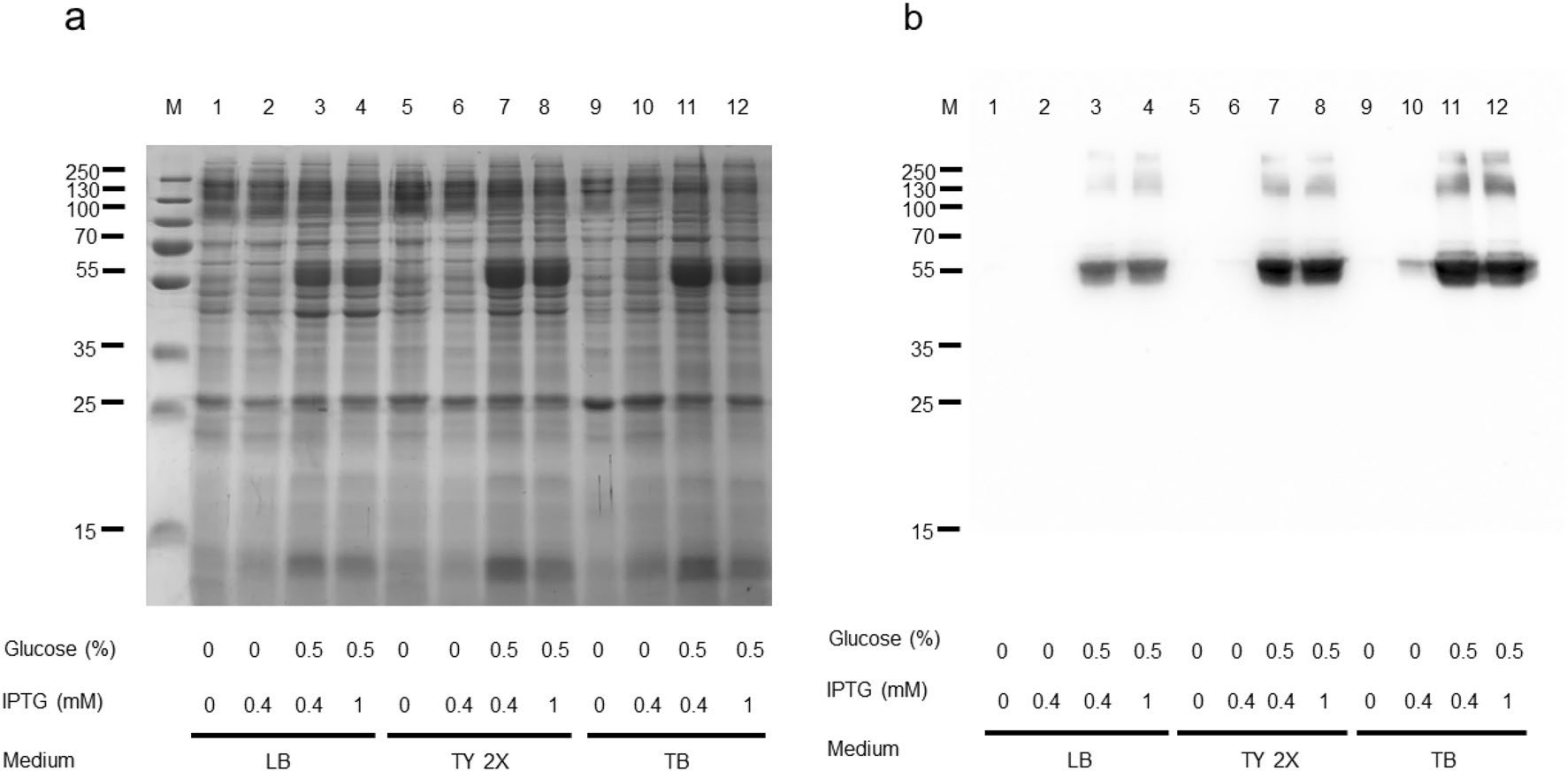
Human SNAT2 expression: the effect of different media *E. coli* BL21(DE3) codon plus RIL was transformed with pET-28a-Mistic-SNAT2-6His and cultured in the indicated media in the absence or presence of 0.5% glucose and in the presence of the indicated IPTG concentrations. a) SDS-PAGE of insoluble fractions after 6 h of growth; M, page ruler prestained plus marker. (b) Western blotting using anti His antibody of the samples loaded as in (a)

SDS-PAGE and Western blotting of the samples (Fig. 1a and b) highlight the crucial role of glucose on SNAT2 expression (Fig. 1a, b lanes 6–9) that was virtually negligible in the absence of glucose (Fig. 1a, b lanes 1–5). The protein production increased with the IPTG concentration (Fig. 1a, b lanes 7–9) with optimal conditions at 0.4-1 mM. The effect of different growth media, TY 2X and terrific broth (TB), was also tested either in the absence or in the presence of 0.5% glucose. The expression level order was: Terrific (Fig. 2a, b lanes 9–12) > TY 2X (Fig. 2a, b lanes 5–8) > LB (Fig. 2 a, b lanes 1–4), and the best expression was obtained culturing the cells in terrific broth in the presence of 0.4 mM IPTG and 0.5% glucose (Fig. 2a, b lane 11).

**Fig. 3:**
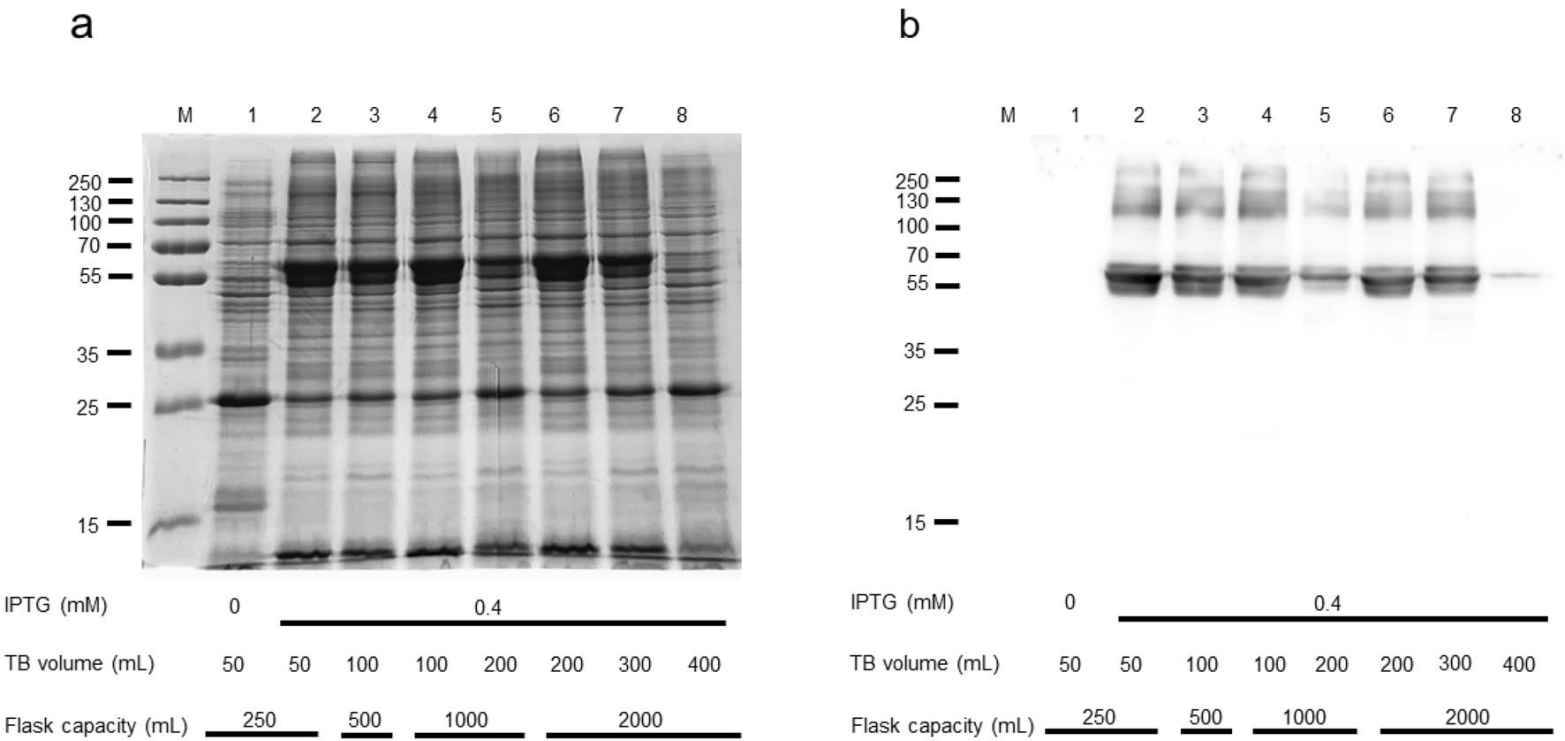
Human SNAT2 expression: the effect of oxygen. *E. coli* BL21(DE3) codon plus RIL was transformed with pET-28a-Mistic-SNAT2-6His and cultured in TB in the presence of 0.5% glucose and 0.4 mM IPTG. (a) SDS-PAGE of insoluble fractions of uninduced (lane 1) and induced cell lysates (lanes 2–8) deriving from cultures in Erlenmeyer flasks with different capacities filled at different levels, as indicated in the figure. M, page ruler prestained plus marker. (b) Western blotting using anti His antibody of the samples loaded as in (a)

These data indicate that Mistic-SNAT2 was not toxic to bacteria. To improve the large-scale production of the protein of interest, we tested the effect of oxygen on protein expression by culturing different volumes of TB cultures in Erlenmeyer flasks with different capacities (Fig. 3a, b). This experimental design allowed us to verify that the best expression conditions were obtained with the lower ratios between culture and flask volumes, indicating that oxygenation may be important for expression (Fig. 3 ab). We then chose the growth condition with 200 mL in 2L flask as the best compromise between optimal expression and total protein amount.

## Purification of the hSNAT2 transporter

**Fig. 4:**
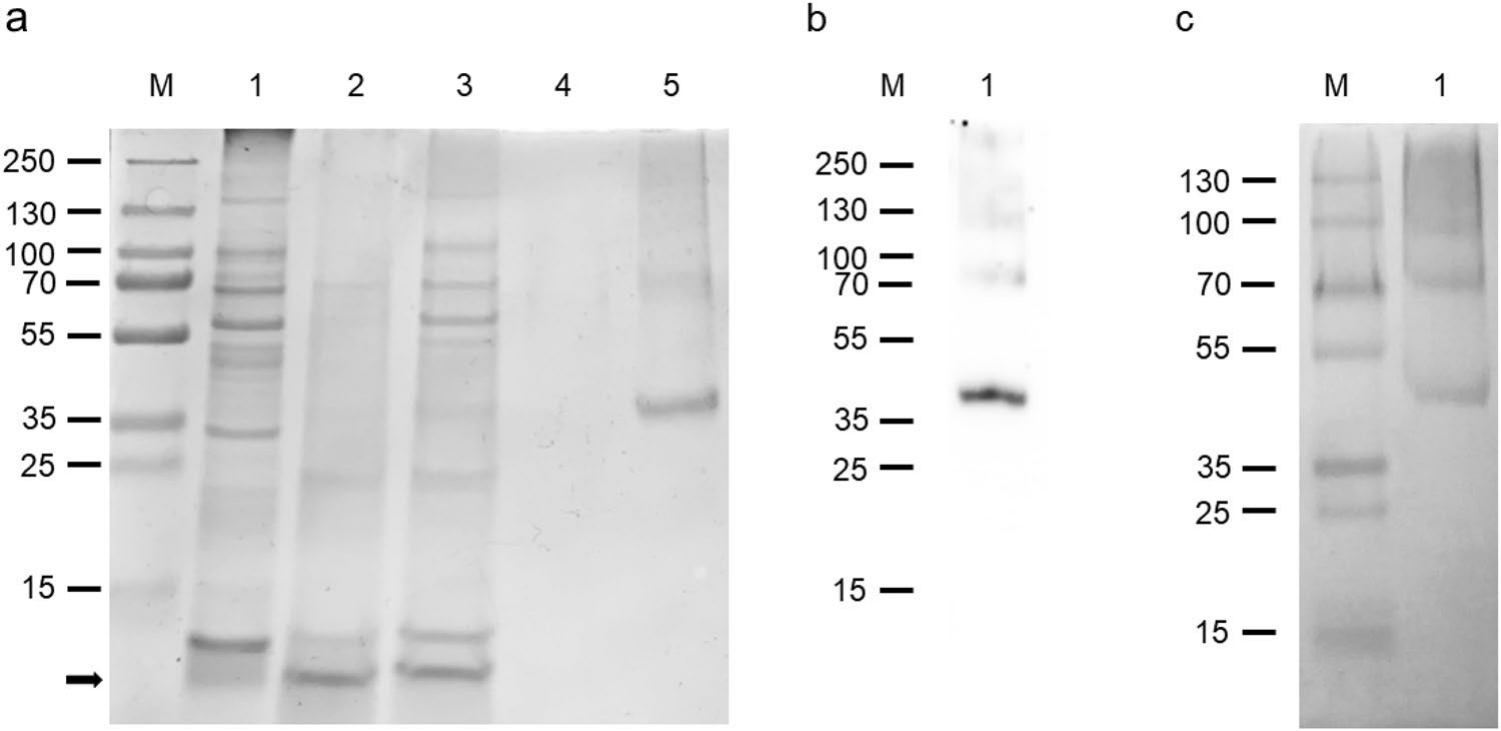
Purification of the SNAT2 transporter. (a) Proteins were separated by SDS–PAGE: lane M, page ruler prestained plus marker; lane 1: sample as in Fig. 3 (a) lane 6 solubilized before IMAC loading; lane 2: flowthrough fraction containing the unbound proteins; lane 3: fraction of the proteins eluted with washing buffer; lane 4: fraction of the proteins eluted with washing buffer added with 15 mM imidazole; lane 5: fraction of the protein eluted with 500 mM imidazole. Mistic protein visible in lanes 2 and 3 is indicated by a black arrow. (b) Western blotting of the same sample loaded in Fig. 4a lane 5. (c) Sarkosyl-PAGE of the same sample loaded in Fig. 4a lane 5

The Mistic-SNAT2 was solubilized under denaturing conditions (Fig. 4a, lane 1) and purified by IMAC by exploiting the 6His tag inserted by the cloning procedure at the C-terminus. During the binding to the resin, thrombin was added to cleave and remove the Mistic tag, which was released in the passthrough and wash fractions (Fig. 4a, lanes 2 and 3, respectively). Following a further wash in the presence of 15 mM imidazole (Fig. 4a, lane 4), hSNAT2 was eluted in the presence of 500 mM imidazole, resulting in a protein band with an apparent molecular mass of 40 kDa (Fig. 4a, lane 5). A very faint band was also detected with an apparent molecular mass of about 75 kDa, i.e., double the monomer molecular mass. Interestingly, by performing the electrophoresis under mild denaturing conditions, that is, by substituting the SDS with sarkosyl [24], the 75 kDa band was more represented than the monomer, indicating the formation of a SNAT2 dimeric form; higher molecular mass aggregates, if any, are less than the dimeric and monomeric forms (Fig. 4c). The yield of purified protein was 60 mg/Liter of cell culture.

This correlates well with previous observations [25] and with the presence in the protein structure of a Leucine repeat motif that was reported to be important for the oligomerization of the SLC6A3 transporter [26]. Further investigation is in the course for assessing the role of the dimeric form in the SNAT2 function.

## Transport function

**Fig. 5:**
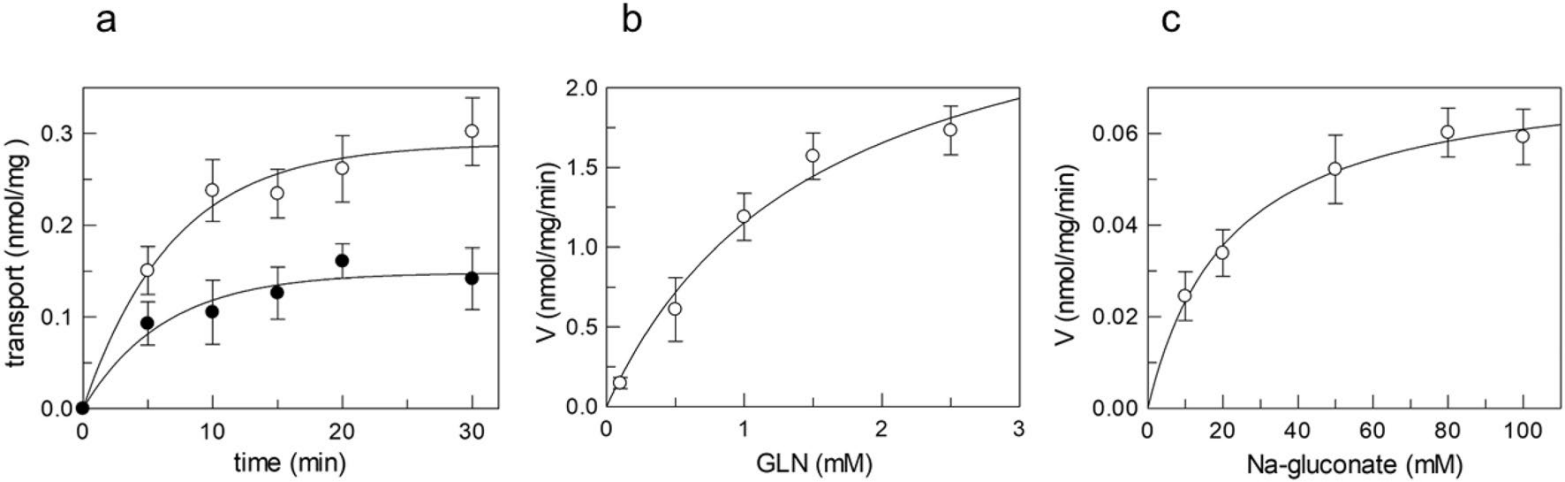
Biochemical characterization of SNAT2 reconstituted in proteoliposomes. (a) Time-course analysis of reconstituted SNAT2. The transport was started by adding 50 μM [^3^H]-glutamine to proteoliposomes in the presence (o) or the absence of 50 mM Na-gluconate (●) and stopped at indicated times. (b, c) Kinetic analysis of the recombinant hSNAT2 reconstituted in proteoliposomes for glutamine and Na-gluconate, respectively. In (b), the transport was measured in 10 min by adding indicated concentrations of external [^3^H]-glutamine to proteoliposomes in 50 mM Na-gluconate. In (c), the transport was measured in 10 min by adding 50 μM [^3^H]-glutamine to proteoliposomes in the presence of the indicated concentrations of Na-gluconate. Data were plotted according to the Michaelis–Menten equation. Experimental data represent the mean ± SD of four independent experiments

To ascertain that the expression and purification procedures led to a functional SNAT2, the protein was reconstituted in proteoliposomes after purification and on column detergent dilution/substitution. Indeed, the ionic detergent sarkosyl, used for solubilizing the insoluble cell fraction (see Materials and Methods), was substituted during the purification procedure by 0.3% DDM. A reconstitution procedure adopted for other transporters was performed with modifications for SNAT2 (see Materials and Methods). After reconstitution, the protein was able to transport ^3^H-glutamine, which was reported as one of the SNAT2 substrates [3]. As shown in Fig. 5a, the activity was time-dependent and could be fitted by a first-order rate equation, typical of a protein-mediated process. Sodium added to the external environment strongly stimulated the transport. This data correlates well with previous data obtained in intact cells and by bioinformatics describing a sodium-dependent feature of this transporter [8, 21]. The stimulation by sodium from the external side is in favor of a right-side-out orientation of the protein in the artificial membrane with respect to the native membrane, indicating that the proteoliposome system mimics the cell membrane layout. Thus, transport kinetics were performed by studying the dependence of the transport rate on glutamine or sodium concentration (Fig. 5b and c).

The curves well fitted a Michaelis-Menten equation in both cases. A Km of 1.54 ± 0.51 mM and a Vmax of 2.92 ± 0.49 nmol/mg/min were derived for glutamine transport, whereas the Km for sodium was 21.78 ± 2.79 mM. The Km for glutamine or Na^+^ is similar to that previously measured in intact cells for the rat ortholog, whose values were 1.65 [8] or between 11 mM (at pH 8) and 52 mM (at pH 7), respectively [27]. The data obtained validate the suitability of the developed protocol.

## Conclusion

A novel procedure of expression and purification was pointed out for obtaining a functionally active SNAT2 protein that can be exploited for further structure/function relationship studies and for validating ligands of SNAT2 predicted by computational high-throughput screening. This last application has a great relevance to human health owing to the physio-pathological implications of this transporter. Moreover, the experimental model will enable to correlate the function of the transporter to its oligomeric state. The relatively high protein yield obtained by this approach may also be a starting point for further optimization in view of structural studies.

## Data Availability

Not applicable.
